# Innate Mechanisms in Selective IgA Deficiency

**DOI:** 10.3389/fimmu.2021.649112

**Published:** 2021-04-26

**Authors:** Jingyan Zhang, Dèlenn van Oostrom, JianXi Li, Huub F. J. Savelkoul

**Affiliations:** ^1^ Cell Biology and Immunology Group, Wageningen University & Research, Wageningen, Netherlands; ^2^ Lanzhou Institute of Husbandry and Pharmaceutical Sciences, Chinese Academy of Agricultural Sciences (CAAS), Lanzhou, China

**Keywords:** selective IgA deficiency, innate mechanisms, T-cell independent switching, TNFRSF13B gene, TACI, Treg, epigenetic imprinting

## Abstract

Selective IgA deficiency (SIgAD), characterized by a serum IgA level below 0.07 mg/ml, while displaying normal serum levels of IgM and IgG antibodies, is the most frequently occurring primary immunodeficiency that reveals itself after the first four years after birth. These individuals with SIgAD are for the majority healthy and even when they are identified they are usually not investigated further or followed up. However, recent studies show that newborns and young infants already display clinical manifestations of this condition due to aberrancies in their immune defense. Interestingly, there is a huge heterogeneity in the clinical symptoms of the affected individuals. More than 50% of the affected individuals do not have clinical symptoms, while the individuals that do show clinical symptoms can suffer from mild to severe infections, allergies and autoimmune diseases. However, the reason for this heterogeneity in the manifestation of clinical symptoms of the individuals with SIgAD is unknown. Therefore, this review focusses on the characteristics of innate immune system driving T-cell independent IgA production and providing a mechanism underlying the development of SIgAD. Thereby, we focus on some important genes, including TNFRSF13B (encoding TACI), associated with SIgAD and the involvement of epigenetics, which will cover the methylation degree of TNFRSF13B, and environmental factors, including the gut microbiota, in the development of SIgAD. Currently, no specific treatment for SIgAD exists and novel therapeutic strategies could be developed based on the discussed information.

## Introduction

Immunoglobulin (Ig) A is produced in intestines at a rate of 3-5 g/day and is present in the blood circulation at a concentration of 2-3 mg/ml (1, 2). Therefore, IgA plays a very important role in immune protection at mucosal surfaces in the respiratory and gastrointestinal tract (3). This production of IgA consumes more energy than producing all the other antibody isotypes together (1). However, individuals with selective IgA deficiency (SIgAD) do not have IgA and lack this major protector (2, 4). SIgAD is generally considered to be the most common primary immunodeficiency and is defined by a decreased level or even complete absence of IgA in the blood while the other antibody isotypes occur at normal levels in children beyond four years of age (3).

However, recently it was shown that components of the innate immune system are crucially involved in this condition and these are detectable already in newborns/very young infants. These new insights may facilitate more early diagnosis and may provide novel opportunities for rationally designed therapeutic strategies for this currently untreatable disease. Moreover, SIgAD is distinguished from other immunodeficiencies as more than 50% of the affected individuals do not show any clinical symptoms, contrary to individuals with other immunodeficiencies (5). Even though SIgAD is so prevalent, the underlying cause of SIgAD and its heterogeneity in expression of clinical manifestations is still unknown (3). These notions illustrate the knowledge gap with respect to this condition and we therefore focused on our current understanding.

## Characteristics of IgA

### IgA1 and IgA2 Subclasses

IgA is the most prominent antibody isotype of the mucosal immune system and while all other mammalian organisms have only one class of IgA, humans have IgA in two subclasses, called IgA1 and IgA2 (6). IgA1 and IgA2 are each encoded by different genes, α1 and α2 respectively, located on chromosome 14 (1, 7, 8). IgA1 is mainly found as a monomer in serum and about 90% of serum IgA is monomeric IgA1 and is additionally found in saliva, mammary glands, respiratory tract and proximal parts of the gastrointestinal tract as a dimer. IgA2 is mainly found in secretions, for example in the gut, saliva and in respiratory mucus, and is mainly found as a dimer (9). The mucosa of the respiratory tract and gastrointestinal tract consists approximately for 60% of IgA2 (10). The main difference in molecular structure between IgA1 and IgA2 is that the IgA2 isotype has a sequence deletion of 13 amino acids in the hinge region, and is therefore more resistant to proteases from pathogens like *Haemophilus influenzae type 1* and *Neisseria gonorrhoeae type 2*, in respiratory and gastrointestinal tracts (11–13). IgA2 lacks these sequences because of the deletion and therefore remains relatively resistant to proteolytic cleavage (1). Monomeric human IgA does not activate complement and is therefore considered anti-inflammatory. However, in conditions like IgA nephropathy there might be a difference in the degree of glycosylation between monomeric versus polymeric IgA while present in immune complexes and this can affect complement activation (14).

### Serum IgA

The monomeric form of serum IgA consists for about 90% of IgA1 and about 10% of IgA2 (12). This serum IgA is produced by long-lived plasma cells in the bone marrow (1). Serum IgA complexed with antigen can bind to receptors on phagocytes specific for IgA (FcαR) and thereby might play a role in the activation of these phagocytes (8). IgA coated particles on neutrophils enhanced the formation of reactive oxygen species (ROS) and neutrophil extracellular traps (NETs) and the secretion of leukotriene B4 (LTB4) enhancing chemotaxis of neutrophils and monocytes (15). After binding of the serum IgA-antigen complex, phagocytes (including monocytes, macrophages and neutrophils) ingest the complex and thereby clear the blood circulation of antigens. Complement activation by IgA antibodies has been previously shown to involve the alternative pathway and the lectin pathway, but not the classical complement pathway (16). In sterile inflammation without the presence of pathogens, complement activation is still implicated with a critical contribution of IgG and not IgA antibodies, to downstream proinflammatory change as complement participates in tissue remodeling, recognition of damaged cells and the enhancement of phagocytosis (17).

### Secretory IgA

IgM+ B cells are present in the Peyer’s patches (PP) and can be activated by antigens, which are delivered by microfold (M) cells lining the PP located particularly in the small intestine (16, 17). Subsequently, these activated B cells switch from IgM+ to IgA+ B cells in a T cell-dependent and T cell-independent manner. These IgA+ B cells leave the PP and enter the blood circulation and by the expression of homing receptors, they migrate to the lamina propria present in the mucosal surfaces of the gastrointestinal and the respiratory tract where they differentiate into IgA-secreting plasma cells. This IgA secreted by the plasma cells at mucosal surfaces is almost exclusively a dimer (in contrast to the monomeric IgA in the blood circulation). Within the dimeric IgA the two monomer IgA molecules are coupled by a joining (J) chain. This J chain is, next to the IgA molecule, produced by IgA plasma cells. With its J chain, the dimer IgA can bind to specific transport receptors (poly-Ig-receptors) on epithelial cells. After binding to the receptor, the entire IgA-receptor complex will be endocytosed and transported intracellularly to the cell surface at the luminal side. Next, the poly-Ig-receptor will be cut after which the extracellular domain of the receptor bound to the IgA molecule, will be released into the lumen of the gastrointestinal or respiratory tract. The receptor part which stays connected to IgA is known as the secretory component and this IgA connected to the secretory component is known as sIgA. This secretory component protects the IgA against proteolytic enzymes which are present in the gastrointestinal and respiratory tract. The dimeric secreted form of IgA (sIgA) consists of IgA1 and IgA2 subclasses (12) and is predominantly found in secretions including saliva, tears, breast milk, colostrum, from the lamina propria of the gastrointestinal, respiratory and genitourinary tracts (18). While serum IgA, like other antibody isotypes, is produced by long-lived plasma cells in the bone marrow, most of sIgA (~95%) is produced locally at the mucosal surfaces (1, 2).

Protective mechanism of secretory IgA primarily comprise exclusion or the protection of epithelium from exposure to harmful antigens (both commensal and pathogenic bacteria) by preventing their adhesion and penetration and exposure of the mucosal immune system to their antigens. This is based on immune complex formation by means of the sIgA Fab fragments (3). Alternatively, sIgA can make complexes in a non-antigenic-specific manner by binding with its Fc part to sugars (like mannose) on pathogens (19). sIgA can neutralize and help to remove antigenic substances as toxins, enzymes, bacteria and viruses in the lumen or for example neutralize viruses hiding in epithelial cells (20, 21).

sIgA-antigen immune complexes can bind phagocytes expressing the Fc-receptor specific for IgA molecules (FcαR) referred to as opsonization which facilitate the antigen uptake and subsequent processing (22). Yet another function of sIgA in the lamina propria is the activation of the complement system *via* the lectin pathway (23). Lastly, sIgA has a transport function by transporting antigens form the lumen to the lymphoid organs, called retrograde transport based on the expression of the transferrin receptor (CD71) on epithelial cells or by binding to Dectin-1 expressed on M-cells (24). Dendritic cells are present in the lamina propria and they are characterized by their uptake, processing and presentation of antigenic fragments to T and B cells. In summary, sIgA is of great relevance in the first line of defense and in protection against most infectious agents.

### Innate Mechanisms in IgA Formation

The mucosal surfaces in different organs collectively form a network in which antigens and regulatory processes are shared amongst the various tissues and organs thereby providing effective immunity and this network is generally referred to as the common mucosal immune system. In utero, this mucosal network is fully developed by 28 weeks of gestation. However, in the absence of an intrauterine infection, this activation does not occur until after birth. The capacity to generate a mucosal immune response develops rapidly in the first weeks after birth and is only fully active upon antigen exposure after the first year of life. Besides exposure to pathogens and commensals bacteria, also diet modulates the immune development during the early lifespan and between 1 and 6 months of age. During this age-related maturation of the mucosal immune system, IgA antibodies increase in serum and secretions like saliva starting between 4 and 6 weeks of age. Also, during the first year of life, the predominant monomeric IgA production switches to sIgA.

The innate and adaptive immune systems are interconnected to provide rapid and efficient protection by responding to danger signals, subsequently eliminating pathogens and by creating highly specific and long-lasting immunological memory in addition to providing tolerance to self. The innate immune system uses germline encoded receptors recognizing conserved pathogen patterns while the highly specific adaptive immune system uses specific T- and B-cell receptors based on recombined gene segments for antigen recognition and memory formation. Adaptive immunity is dependent on the innate response for the initiation and direction of the response, illustrating the integration of these two systems to ensure the efficient generation and regulation of protective immune responses (10, 25). Dendritic cells are members of the innate immune system but are also the most efficient antigen-presenting cell able to stimulate T cells to respond to a specific antigen, thereby bridging these two parts of the immune response (25).

An important insight is that isotype switch recombination to IgA was shown to occur in a T-independent manner as switch promoting cytokines (e.g., BAFF, APRIL) released from dendritic cells and innate lymphoid cells (ILC). In addition, it has been shown that most of this T-cell independent IgA is part of the natural antibody repertoire that is constantly produced in the absence of antigenic stimulation and are polyreactive and of low affinity. Therefore, such antibodies can be considered to be part of the innate immune system. It is remarkable that this T-cell independent IgA is produced in the small rather than the large intestine where the microbial load is highest (26). The innate lymphoid cells (ILCs) are a group of cells in the intestinal mucosa that play important roles in inducing immunity and inflammation (26). The ILC3 subset of cells share the presence of the RORγt+ transcription factor and by producing IL-22 ensure epithelial barrier integrity. IL-22 also increases the secretion of antimicrobial peptides and together these activities prevent the adherence and induction of epithelial inflammation by intestinal microbes. ILC3 also release survival factors for B-cells like BAFF, APRIL and CD40 Ligand, and thereby contribute to the T-cell independent mucosal IgA antibody formation (27).

The gastrointestinal tract harbors most of the commensal bacteria and the local mucosal immune system is considered crucial in the maintenance of intestinal homeostasis. SIgA plays an important role in this process based on its immune exclusion mechanism, but also by regulating the numbers and diversity of intestinal bacterial communities. In mice it was shown that lack of sIgA induced the uncontrolled expansion of anaerobic bacterial species (28).

## Characteristics of Selective IgA Deficiency

### Infant Disease Expression

Currently, over 180 primary immunodeficiency diseases have been described of which antibody disorders constitute about 55% (29). SIgAD is the most frequently described form that is partly genetically based and therefore considered a primary deficiency, although it can also be secondary as it can be induced by exposure to drugs or the result of other diseases. However, young children after 4 years of age might have low serum IgA levels for their age and as a result are classified as “partially deficient” or having “probable” IgA deficiency. Such condition is generally temporarily and has been found in a few adults. In children such a condition is more frequent and likely due to the delayed formation of IgA (30).

The placenta was widely considered sterile, although this view was challenged. However, it was recently shown that the placenta does not contain an independent microbiome although it can harbor certain pathogen resulting in an exposure of the fetal immune system to some microbes before birth (31). The immune system in a newborn contains all the major cell populations of T-cells and B-cells but these are still antigenically naïve. Upon antigen stimulation these cells do not yet respond in a full functional way as B-cells are compromised in their antibody-secreting potential, T-cells are not fully responsive, and the phagocytosis and migratory capacity of innate immune cells is still poor. Dendritic cells are responsive to antigens from microbial pathogens and dietary compounds, but upon exposure mature into suppression of inflammation rather than immune activation (15, 21).

As a result, immune responses to infections are insufficient and this puts the infant who is suddenly confronted with a high microbial load upon birth at risk (17). The combination of fully developed epithelial barriers, an active innate immune system and the presence of protective maternal antibodies for the first 6 months of age collectively protect the infant against the sudden exposure to microbes. In Westernized countries, nowadays newborn children are less confronted with microbial infections compared to the past and compared to children in developing countries (28). According to the hygiene hypothesis these children develop fewer Th1 mediated responses that are protective against microbial infections and mediate the early inflammation reflecting the start of the innate part of the immune response. This reduced proinflammatory response may facilitate colonization with a commensal microbiome but together with the impaired Th1 cytokine production (e.g. IFN-γ) and the diminished cell-mediated immunity, renders the newborn more vulnerable to infection. However, most infants survive this period without illness due to intact innate immunity and maternally transferred immunoglobulin G (IgG) (15, 30).

SIgA antibodies have the capacity to control intestinal antigens by complexing bacterial antigens for removal or uptake through retrograde transport (24, 25). Even in non-SIgAD newborns lack sufficient IgA antibodies in the gut in early life and maternal antibodies do not contain IgA antibodies; these IgA antibodies appear only at several months after birth when plasma cells are functional in the mucosal tissue. At that time tight junction formation is not complete and the epithelial barrier is not yet fully developed permitting also passive uptake of bacterial antigens from the lumen (17, 18, 20). When sIgA is deficient in early life luminal proteins and bacterial products or bacterial fragments escape this IgA-mediated control and may end up in circulation (29–31). Similarly, also in breast milk sIgA antibodies are present and these ensure complexation of bacteria in the gut of the newborn infant (32). Lack of these IgA antibodies in mothers suffering from SIgAD increases the exposure to dietary allergens as has been shown for cow’s milk allergy (32). For cow’s milk allergy it is suggested that this enhances the risk to develop food allergy in infants. Maternal consumption of food products does sometimes result, albeit not consistently, in breast milk containing the relevant dietary allergens. Moreover, sometimes such dietary allergies might be accompanied by lower levels of IgA antibodies in milk. Again, this is not consistent as this often pertains to IgA2 rather than total IgA antibodies that often are of different epitope specificity compared to plasma IgA antibodies (33). Upon successful immunotherapy the levels of cow’s milk and egg-specific IgA2 increase in the plasma, but not necessarily in breast milk, and this IgA2 is mechanistically linked to the induction of tolerance (34). Therefore, there is clear need for direct determination of dietary antigens in breast milk in order to characterize their role in the induction of oral tolerance induction in infants (35)

### Diagnosis

Individuals that suffer from chronic or recurrent infections, chronic diarrhea, different types of autoimmune diseases or any combination of these and are older than 4 years of age are usually tested for selective IgA deficiency. However, even in infants beyond 6 months of age and that show clinical signs of recurrent upper and lower respiratory tract infections from encapsulated bacteria should be evaluated for IgA deficiency. The diagnostic procedure should include the measurement of serum antibodies of IgM, IgG and IgA class and a complete blood cell count complemented with the measurement of peripheral blood B cells, T cells, and NK cells. The current practice of only measuring serum IgA levels in children beyond 4-6 years of age should therefore be adapted when the infection history is consistent with SIgAD (36).

About 20% of SIgAD patients might also show low levels of IgG2 and/or IgG4 in their serum. It was suggested that the IgG2 deficiency in combination with SIgAD determines whether clinical illness will be manifested together with recurrent infections, autoimmune disorders, atopy or malabsorption (37, 38). The diagnosis of SIgAD is therefore often accidental when patients are screened for the presence of other diseases like allergies or autoimmune diseases (34). The SIgAD diagnosis is usually based on the measurements of IgA concentrations in the blood of a patient older than four years being below 0.05 mg/ml (5 mg/dL) while levels of IgG and IgM are normal. Normal adult’s IgA levels are 0.09 - 4.5 mg/mL (90-450 mg/dL). Hence, there is no clear suspicion of other causes of hypogammaglobulinemia and T cell defects while normal IgG antibody response to vaccines occur (39). However, some individuals with confirmed SIgAD may also have deficiency in IgG2, IgG4 and IgE (40, 41). In routine diagnose nephelometry is widely used which has a threshold of 0.07 mg/ml and for that practical reason the value of 0.07 mg/ml IgA is often reported as the cut-off value.

However, there is no consensus on this threshold value. Such values are dependent on the sensitivity of the assay employed and range from 0.07 g/L for nephelometry, 0.05 g/L for radial immunodiffusion plates, and 0.0016 g/L for hemagglutination inhibition techniques. When IgA is detectable but reduced with more than 2 SD compared to the normal range of age-matched healthy controls, this condition is called partial IgAD. This most frequently occurring in children under 4 years of age. At the age of 14 years, about half of these children have reached normal IgA levels and for that reason this condition is referred to as transient IgAD, although some remain low and may progress to common variable immunodeficiency (CVID). Therefore, in children aged 6 months to 4 years with low serum IgA levels these measurements should be repeated at 4 years of age to confirm the formal diagnosis of SIgAD. Importantly, nearly all patients with SIgAD show a deficiency in both secretory IgA1 and IgA2 in their mucosal external secretions, although these IgA subclasses are not routinely measured (42).

Surprisingly, when SIgAD patients are receiving transfusions with blood or antibody-containing blood products, there are a small number of SIgAD patients that subsequently produce IgG or IgE antibodies against the IgA antibodies present in these transfusions. These antibodies can provoke adverse transfusion reactions of which the incidence is about one in 20,000 to 47,000 transfusions (43). Some of these SIgAD patients (around 20%) that develop high anti-IgA levels might even develop severe anaphylaxis. Children with SIgAD that require transfusion with blood or blood products, should thus receive products without containing IgA antibodies. Such products were used in the past and were found to be able to induce transfusion reactions and maybe even severe anaphylactic reactions, although there is debate about the relevance of this in literature (43–46). Currently, washed red blood cells and plasma or platelets from known IgA deficient donors are used to avoid this risk (44, 45).

When mothers who have normal IgA levels, give birth to children that suffer from SIgAD these newborns still will have detectable serum IgA antibodies and even more so when mothers are breast-feeding. Breast milk contains IgA antibodies, and these can protect the gut of the infant against pathogens. However, when mothers are diagnosed with SIgAD their newborns will be devoid of serum IgA and these observations indicate that this SIgAD condition is already present at birth (47). Formally, the definition of SIgAD is based on the measurement IgA antibodies in the serum while the majority of IgA antibodies are present in the mucosal tissues where IgA levels cannot be determined. For that reason, sIgA levels in feces might be representative for the colon (48). In the feces of breast-fed infants, the amount of IgA is in the first 2-4 weeks of life much higher than in formula-fed infants that have undetectable levels. Gradually at 6 months of age the fecal IgA levels converge between these two groups of infants and by 1-2 years of age, when weaning is completed, adult levels of fecal IgA are reached. Conversely, in serum the levels of IgA reach adult levels only by the age of 8 years (47, 49). Remarkably, the advice to diagnose SIgAD in children beyond 4 years of age is based on serum IgA levels only. Therefore, there is a pressing need to design a diagnostic procedure to rapidly identify these children at younger age and to relieve them of the burden of recurrent infections and the predisposition to develop other associated diseases later in life.

### Epidemiology

The prevalence of SIgAD is approximately in 1:700 individuals worldwide with a range from 1:134 to 1:18,500 depending on the population analyzed (46). However the prevalence of SIgAD varies with different ethnic background and global location and is highest in European descendants and Caucasians and lowest in oriental and Asian populations (2, 50). The overall prevalence of SIgAD differs between 1:173 (in Sweden in 2009) (51) and 1:3024 individuals (in the USA in 1969) (52). Whereas the prevalence in Asia differs between 1:3230 individuals (in China in 2010) (53) and 1:14800 individuals (in Japan in 1986) (54). However, the prevalence of SIgAD may be underestimated because there are many SIgAD individuals without having clinical symptoms (2). The variation in the prevalence of SIgAD may also result from the use of non-uniform study designs in the different countries by including individuals with a specific illness and thereby not reflecting the prevalence of the entire population (52). In addition, the criteria used for SIgAD diagnosis are not uniform as some studies use a concentration of <0.07 mg/ml to diagnose SIgAD whereas other studies use <0.05 mg/ml or even <0.01 mg/ml (52). Also, SIgAD occurs more in countries with high rates of consanguineous marriage (55). A rapid antibody test has become available that allows for population-based screening to diagnose selective IgA deficiency already in young children (49).

### Genetic Background of Selective IgA Deficiency and Associated Clinical Manifestations

SIgAD can occur sporadically but familial inheritance also does occur. However, no distinct Mendelian inheritance pattern is found as both autosomal recessive, autosomal dominant and sporadic inheritance were described (56, 57). This heterogeneity in inheritance can be the result of genetic defects in different genes which are inherited in different ways, *e.g.* deletions in IgA1, IgA2 and IgG heavy-chain genes on chromosome 14. Factors associated with the prevalence of SIgAD also includes the family history as it was estimated that first degree relatives have a 38-fold higher prevalence rate then unrelated individuals (58). Approximately 20% of SIgAD are inherited based on pedigree analysis (58). Furthermore, the risk of getting SIgAD is 50-fold increased for first degree family members of patients with SIgAD (55, 56, 59–61). The chance of getting IgA deficiency from an individual’s parents is higher (20.1%) when the mother has IgA deficiency compared to when only the father has IgA deficiency (5.8%) (62). This difference could be explained by paternal imprinting of IgA deficiency susceptibility gene(s) and indicates the involvement of epigenetics in the development of SIgAD.

SIgAD is a disease where multiple different genetic abnormalities can be causally linked (7, 63). In general, however, normal numbers of B cells are found in individuals with IgA deficiency (5). Furthermore, the constant α1 and α2 genes are in most cases normally expressed and do not contain mutations in their coding regions (64). Together, this suggests that there must be a defect in the production or secretion of IgA (65, 66). Additional genetic abnormalities described in literature and involved in the development of SIgAD are described here.

#### HLA

The gene complex that encodes the major histocompatibility complex (MHC), in humans known as the human leukocyte antigen (HLA) complex, is located on chromosome 6. Certain HLA haplotypes belonging both to MHC class 1 and MHC class 2 genes, especially HLA-A1, HLA-B8, HLA-DR3 and HLA-DQ2 (collectively known as the 8.1 haplotype) (67), is the most frequently found haplotype in individuals with IgA deficiency and could be a risk factor for SIgAD in northern parts of Europe (49). Yet other haplotypes, including B14 and DR1 are more prevalent in SIgAD patients in southern Europe and HLA- DR15, HLA-DQ6, are associated with an absence of IgA deficiency and might thus be protective against development of the disease (68–70). In addition, the haplotype HLA-DR2 is also thought to reduce the risk of the development of IgA deficiency (71). Also MHC class III regions were suggested to contain SIgAD susceptibility genes. A detailed genetic analysis, however, showed that the influence of genetics is only modest, with a heritability of 0.36, and therefore other genes, epigenetic influences of environmental factors or of relevance in SIgAD development (72).

#### Chromosome 18

SIgAD associations were found with a major predisposing locus on chromosome 18 and structural changes including deletion of the short or the long arm or the presence of a ring-like structure of this chromosome are associated with SIgAD When using cytogenetic and molecular analysis of the karyotype of SIgAD patients no consistent loss or rearrangement of chromosome 18 material could be found and therefore the prevalence of these abnormalities in individuals with SIgAD remains unclear (73, 74). For example, Burgio et al. (75) found ring chromosome 18 in one of the 50 children with SIgAD, while Taalman et al. (76) found one ring chromosome 18 in most children with SIgAD. However, it is also observed that some SIgAD individuals have deletions in both the long and the short arm (77), which indicates that there is probably not one single gene on chromosome 18 that is affected (78). However, which specific genes on chromosome 18 are associated with SIgAD is yet unknown. Patients with karyotypic abnormalities on chromosome 18 that corelate with low or absent IgA levels are often detected soon after birth as they display multiple developmental defects and clinical manifestations reflective of SIgAD. As described above, in infants and young children serum IgA levels are difficult to interpret as they display variable and individual developmental trajectories towards reaching normal values. Due to this heterogeneity in the kinetics of reaching normal IgA levels it will remain difficult to identify relevant susceptibility loci for SIgAD on chromosome 18.

#### TACI, BAFF, APRIL, TSLP in CVID and SIgAD

SIgAD is found more often in individuals that have family members with common variable immunodeficiency (CVID) (34) which is a condition characterized by reduction in serum IgG and IgA levels and in half of the individuals also a decrease in IgM levels (WHO report, 1995) (79). CVID is an example of a primary immunodeficiency which is in most cases based on a polygenic disorder (80). The observation that both SIgAD and CVID can occur in families’ points towards a shared genetic defect based on shared susceptibility loci and alleles. As a consequence a common pathogenesis might be present resulting in a similar spectrum of deficiencies in IgG subclasses, and the gradual development of SIgAD into CVID in the same affected individuals (81). Because of these features, it is suggested that CVID and SIgAD might share a (partially) similar genetic background (58, 82–84).

**Figure 1:**
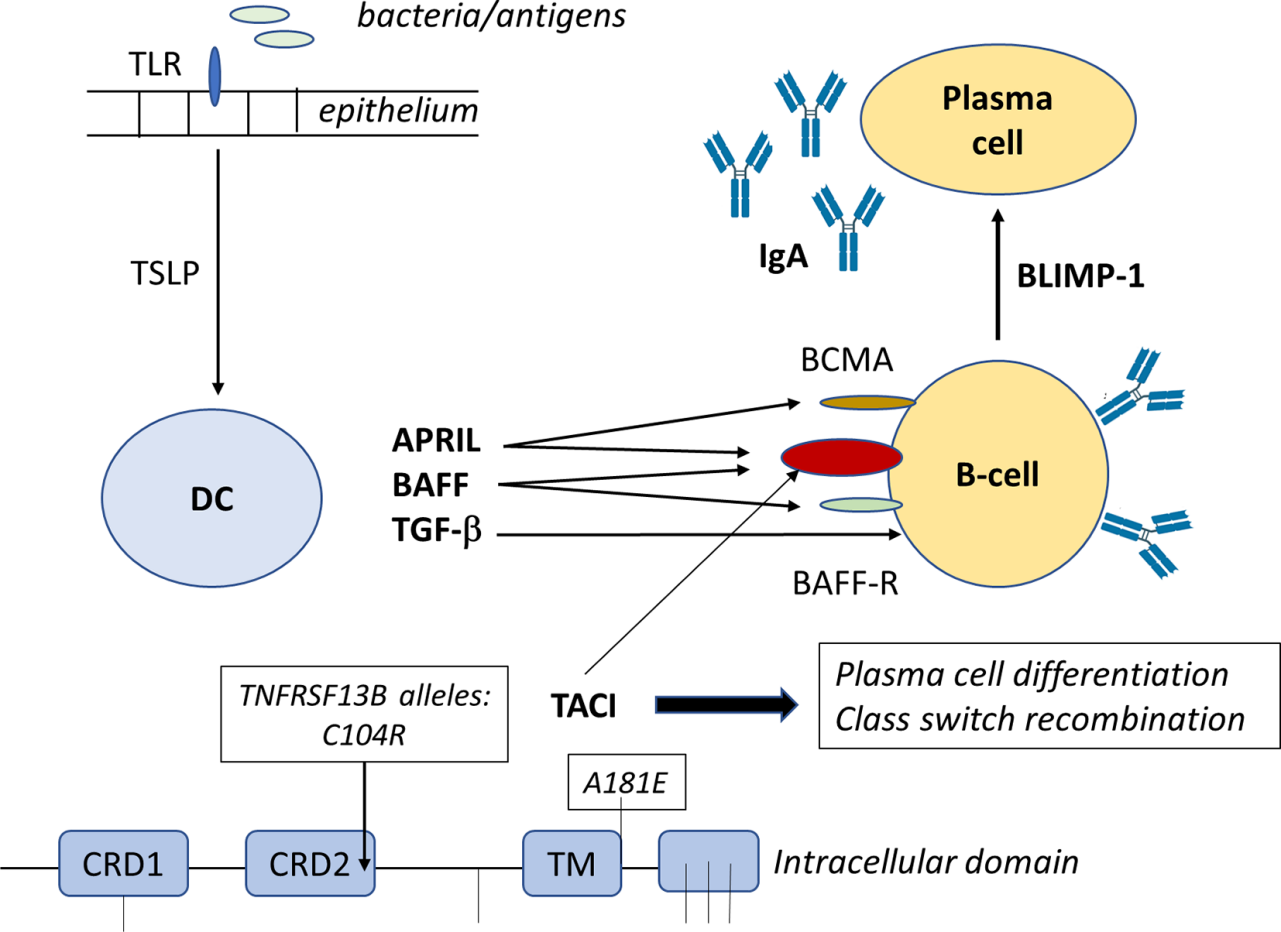
BAFF and APRIL bind to BAFF receptor (BAFFR) and B−cell maturation antigen (BCMA), respectively. BAFF also binds to BCMA. Both BAFF and APRIL bind to TACI receptor. Engagement of BAFFR by BAFF delivers survival signals and class switch recombination (CSR) to peripheral B cells, while engagement of BCMA by BAFF or APRIL delivers survival signals to plasma cells, but has no effect on CSR. BAFF-R stimulation sequesters TRAF, which activates the formation and nuclear accumulation of RelB:p52 complex driving the survival of the B-cells.

**Figure 2:**
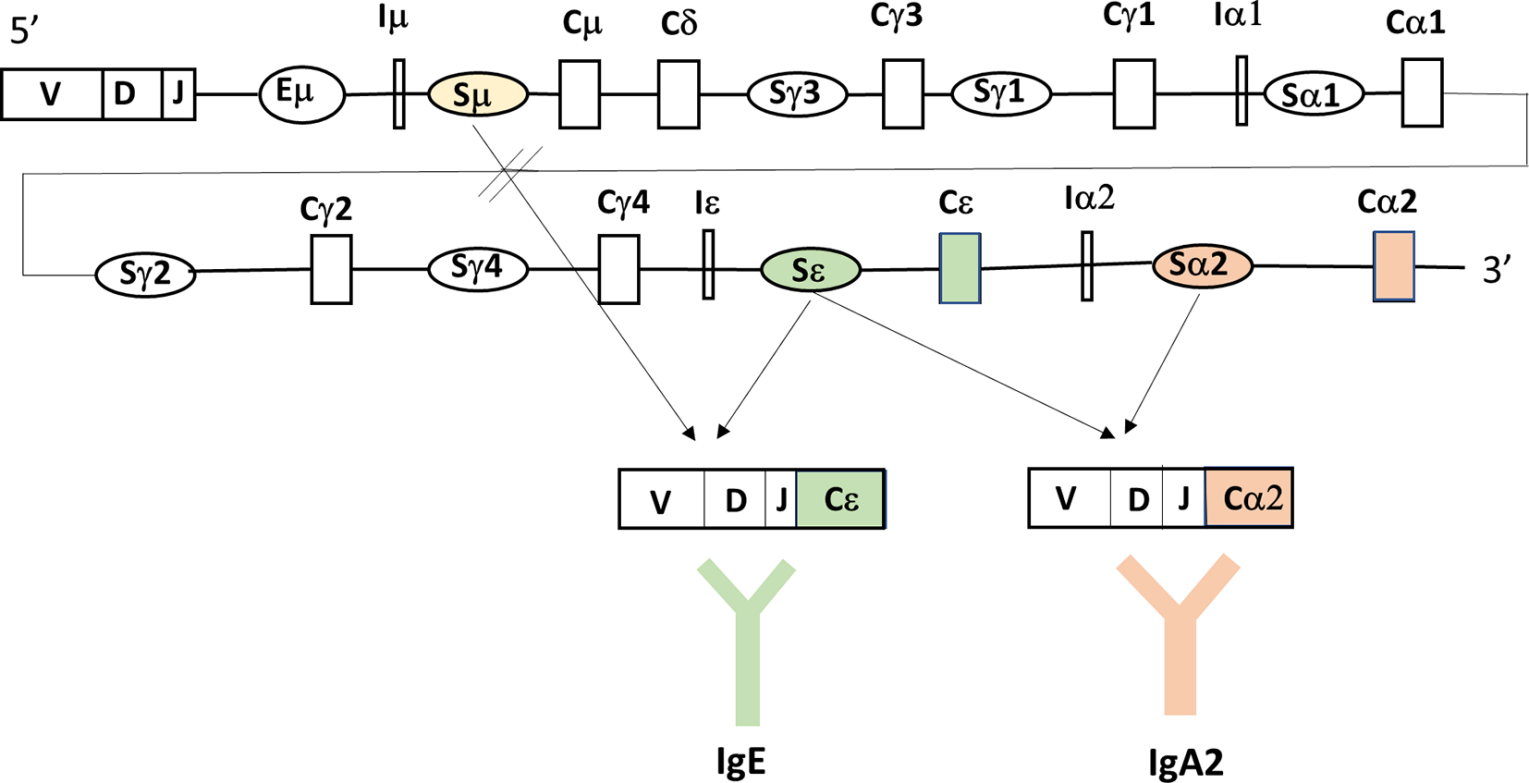
Bacterial fragments or luminal antigens taken up by the gastrointestinal epithelium through TLR-mediated recognition, activate the epithelium to release factors like TSLP that are taken up by dendritic cells. These DC will secrete cytokines like APRIL, BAFF and TGF-β that will impact on mucosal B-cells. APRIL and BAFF will interact with B-cell receptors as explained in **Figure 1**, while TGF-β interacts with receptors to activate Smads (small mothers against decapentaplegic) that are important regulators of B cell responses, including the formation of BLIMP1 that promotes B-cell terminal differentiation to IgA-secreting plasma cells. The TNFRSF13B on chromosome 17, encodes TACI receptor that signals through different transcription factors like NFAT, AP-1, and NF-kappa-B which then modulate B-cell activities, including (T-cell independent) class switch recombination (**Figure 3**), and plasma cell differentiation. In the TNFRSF13B gene contains multiple mutations (indicated by lines) with C104R and A181E being the most frequently occurring ones and this results in altered level of TACI receptor expression and function of the B-cells.

T-cell independent IgA switching is facilitated by the presence of cytokines like BAFF (B cell activating factor) and APRIL binding to their relevant receptors TACI (transmembrane activator and calcium-modulating cyclophilin ligand interaction), BCMA (B cell maturation- antigen) (for both BAFF and APRIL) and BAFF-R (BAFF-receptor) (for only BAFF) (**Figure 1**). As a consequence, IgM+IgD+ naïve B cells switch to IgG and IgA producing B cells (27, 85–88). The link between tumor necrosis factor superfamily member 13 (TNFSF13) and serum IgA concentration was first described in a genome-wide association (89). Mutations in genes coding for ICOS, CD19, BAFF-R and TNFRSF13B (encoding TACI) have been found in 10-15% of SIgAD patients (90, 91). Deficiency in TACI has been found in 10% of SIgAD patients which affects B-cell maturation as TACI is a tumor necrosis factor receptor family member expressed on peripheral B lymphocytes and plays a role in their switching from IgM to the secretion of IgG, IgA and IgE antibodies. In addition, TACI stimulates the formation of memory B-cells that secrete switched isotypes that have undergone affinity maturation (**Figure 2**). Mutations in the TNFRSF13B gene are indicative of CVID although healthy family members and unrelated controls were also reported to carry such mutations, including C104R, 204insA, and A181E. Therefore, since heterozygous TACI mutations occur in healthy controls and clinical presentation ranges from unaffected to severe immunodeficiency, these TACI mutations are not validated in having direct functional consequences. This shows that other genetic alterations may add to the clinical severity of individual phenotypes (92). About 31.5% of the mutations in TACI are represented by C104R which affects the extracellular domain of TACI and thereby abolishes the ligand binding with BAFF. B-cells of SIgAD patients carrying the TNFRSF13B mutations express this mutated TACI but to the same level compared to B cells from healthy controls. On the other hand, these patients show elevated serum TACI but not BAFF levels. Collectively, these observations suggest that mutations in TNFRSF13B could induce CVID and IgA deficiency. Until now TNFRSF13B mutations are not related to clinical manifestations as these mutations do not occur in all CVID and SIgAD patients.

Moreover, these mutations were not linked to the severity or frequency of infections, lymphoproliferation, or autoimmunity in these patients. Therefore, TNFRSF13B mutations might be more related to disease susceptibility rather than being the cause of CVID and SIgAD disease (93). For T-cell independent IgA switching antigen-sampling DC can be modulated by innate stromal factors, including thymic stromal lymphopoietin (TSLP). TSLP is an IL-7–like cytokine that is produced in response to bacterial contact and amplifies mucosal IgA responses by simulating DC production of IL-10, BAFF, and APRIL (94). Polymorphisms in the TSLP gene were described with two SNPs Rs2289276 and Rs2289278. It is as yet unknown whether there is an association between TSLP or the two SNPs and the susceptibility for selective IgA deficiency (95).

#### Additional Genes

More genes are suggested from a genome-wide association study to be related to SIgAD development, including the interferon induced with helicase C domain 1 (IFIH1) and C-type lectin domain family 10, member A (CLEC16A) (68). IFIH1 is an innate immune receptor with the gene located on chromosome 2q24, and which plays a major role in sensing viral infection and in the activation of antiviral responses. CLEC16A is a gene on chromosome 16p13, which encodes a C-type lectin and plays a role in mitochondria quality control. Recently, two other single-nucleotide polymorphisms were implicated in SIgAD: one is located upstream of the gene SAM and SH3 domain containing 1 (SASH1) and the other downstream of the CD30 ligand (CD30L) (72). However, the relevance of these genes in SIgAD remains to be established. Overall, these studies showed suggestive evidence for association with certain susceptibility genes and hence, no causative conclusions can be made yet.

## Different Clinical Manifestations of SIgAD

### Heterogeneity in Clinical Symptoms

SIgAD can be based in some individuals on both cytogenetic defects and monogenic mutations with consequences in the innate immune system. In addition, SIgAD is sometimes associated with defects in phagocytosis capacity and complement activation and thereby shows that SIgAD can be associated with the development of combined immunodeficiencies. The individual variation in SIgAD symptoms can be further explained by the finding of genetic polymorphisms in IL-10 and IL-6 genes. Remarkable about SIgAD is the fact that there are substantial numbers of individuals (60-75%) that do not present clinical manifestations, while others do (2, 5, 93). Moreover, those individuals that do show clinical symptoms have very variable symptoms. Because of this variability in symptoms, a classification based on five different clinical phenotypes has been proposed: asymptomatic (60% of the individuals with SIgAD), mild infections (12%), severe infections (2%), allergy (15%) and autoimmunity (11%) (96, 97) (see **Table 1**).

**Table 1:** Summary of the clinical manifestations in individuals with SIgAD.

Characteristic	Percentage (%)*	References
IgA concentration < 0.07 mg/ml	100%	2, 39–43
Asymptomatic	60%	2, 62, 98–100
Mild infections	12%	39, 40, 98–102
Severe infections	2%	101–105
Allergies	15%	34, 99, 106–117
Autoimmune disorders	11%	54, 61, 99, 118–121
Cancer	4.8%	101, 112–124

*Based on reference 100.

In asymptomatic SIgAD individuals, it is suggested that the loss of IgA antibodies can be (partially) compensated by other components of the immune system, like IgM antibodies in the mucosal tissue or higher levels of IgG in circulation (30). Individuals with SIgAD that do have clinical symptoms mainly present recurrent infections in the upper respiratory tract in addition to otitis media, sinusitis, bronchitis, pneumonia, and gastrointestinal infections, but these are mostly minor infections that generally do not cause serious complications (7, 12–102). Individuals with recurrent and more severe infections have also infections in the lower respiratory tract (82). Individuals with the severe phenotype do not only have more severe recurrent infections, which can cause permanent damage, but also the frequency of autoimmune diseases is often increased, due to their age-related chronic infections that can even develop into more severe CVID disease (52, 91, 103). Autoimmune conditions may also occur in 25% to 35% of those with IgA deficiency (104, 105). Some of the autoimmune diseases that are more common in SIgAD, include: type 1 diabetes mellitus, rheumatoid arthritis, thyroiditis, systemic lupus erythematosus and celiac disease (104–106, 118, 119, 125, 126). The most frequent complication in SIgAD individuals is the development of chronic diarrhea and malabsorption (96, 97, 113).

Individuals with a severe SIgAD phenotype can have an increased frequency of tumors (30, 120). Individuals with SIgAD probably do not have a general increased risk of developing cancer (118) as only some specific cancers, like adenocarcinoma of the stomach, lymphoma and gastrointestinal cancers, are more prevalent in individuals with SIgAD (96, 119, 120). Other specific cancer types found in individuals with SIgAD include colon carcinoma, ovarian cancer, melanoma, lymphosarcoma and thymoma (122, 127). Overall, the prevalence of cancer in individuals with SIgAD is not very high (123, 128). For example, Shkalim et al. (100) followed 63 Israeli children with SIgAD for 10 years and found that cancer developed in 3 of these children, resulting in a prevalence of 4.8% (see **Table 1**) (100). In summary, a great heterogeneity exists in the clinical manifestations of individuals with SIgAD.

### Allergies and Autoimmunity

Allergies are occurring in about 10% to 15% of those affected with SIgAD and the most frequently occurring clinical manifestations are allergic conjunctivitis, rhinitis, urticaria, eczema, food allergy and asthma (7, 93, 105, 107–114, 124, 129, 130). However, there are also studies that have not found such an increased frequency of allergies in individuals with SIgAD (51, 63, 75, 78, 107, 108, 110).

**Figure 3:**
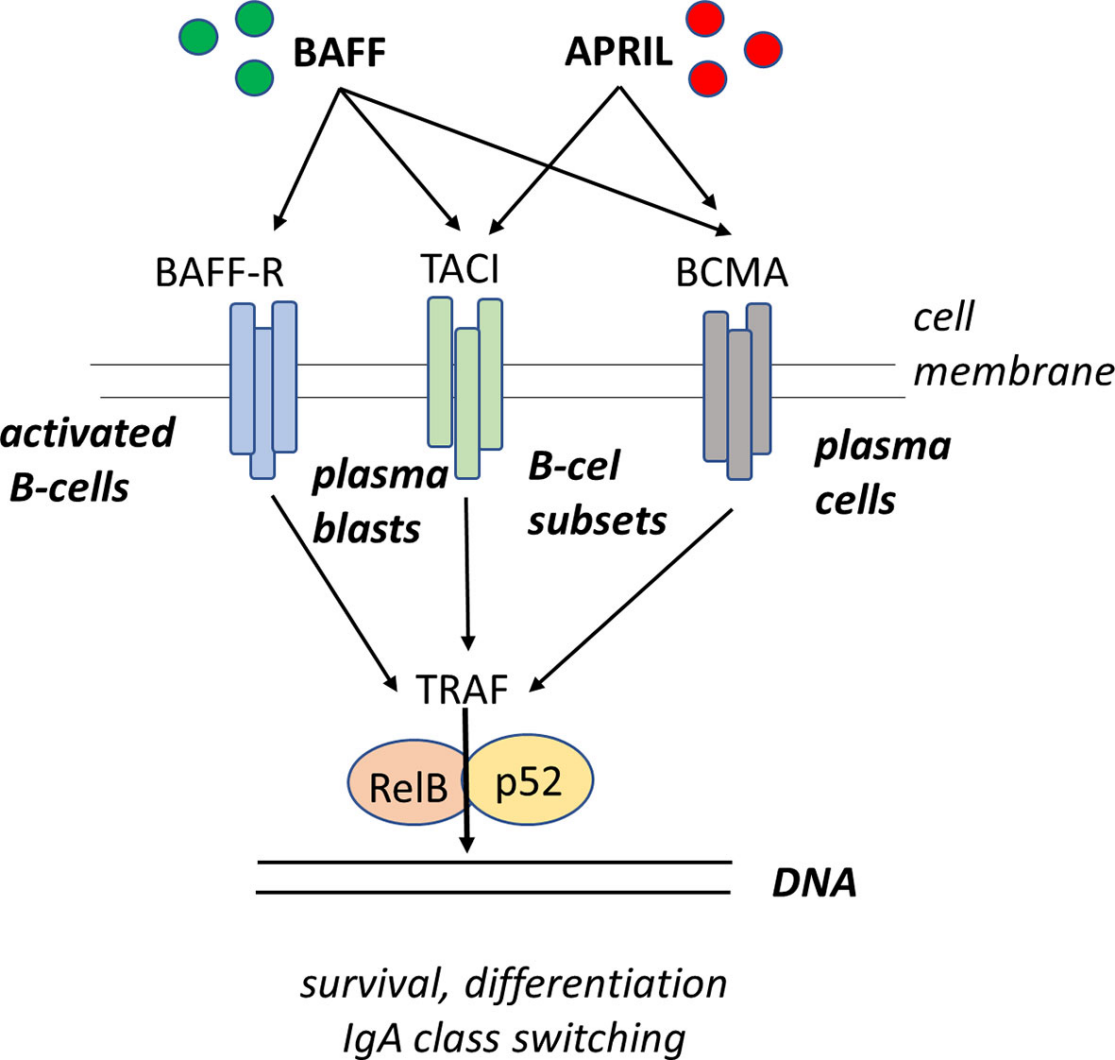
Sequential class switch recombination (CSR) from IgE to IgA2 in activated B-cells. The immunoglobulin heavy chain locus contains a rearranged variable (V) diversity (D) joining (J) exon encoding the antigen-binding domain of the antibody molecule. Upon activation, the B-cell receives specific signal for isotype switching whereby the switch region upstream of the cμ locus (Sμ) integrates with the switch region Sε upstream of the Cε gene segment. As a result, the B-cell produces productive mRNA transcripts that give rise to the formation of IgE molecules. Subsequent exposure to other environmental factors (including TGF-β, APRIL, or BAFF) drives a further CSR event whereby the B-cell fuses the Sε segment to the Sα2 segment, leading to a productive switching to IgA2. As the switching process occurs from 5’ to 3’ and all isotype gene segments that are skipped will be deleted upon the next cell division an IgE-secreting B-cell has only one option left for subsequent CSR which is to move to IgA2.

There is still controversy in the literature on the relevance of IgA antibodies in the protection against the development of allergy. IgA is a mucosal isotype as this is where it is mainly produced, and this IgA has the function of preventing the induction of a local allergic inflammation. It has been shown that serum IgA levels do not correlate with locally produced mucosal IgA antibodies (109, 130). When suffering from allergic rhinitis or allergic asthma, the measurement of allergen-specific IgA antibodies in mucosal secretions including saliva and induced sputum, respectively, appears to be of limited values unless these measurements complement the determination of allergen-specific serum IgE levels. Nevertheless, recent reports showed that salivary IgA correlates with protection against the expression of clinical manifestations of allergy (108). As discussed above, complementing the measurement of IgA by determining levels of IgA1 and IgA2 might extend the relevance of IgA in allergy as especially IgA2 is an isotype associated with mucosal tolerance (112). An attractive hypothesis would be to induce isotype switching in IgE producing B cells of allergic individuals as these have only one possibility to switch to IgA2 allowing allergic individual to become tolerant (130) (**Figure 3**).

The absence of IgA in SIgAD reduces its protective role on mucosal surfaces by preventing the passage of allergens over the epithelium and allowing them to get into contact with the local mucosal immune system. Hence, there is an increased likelihood of sensitization against antigens and for example an increased chance of respiratory tract infections and asthma (113, 114). Next to an increased frequency of allergic disorders, an increased frequency of autoimmune disorders was found in individuals with SIgAD. There are different hypotheses that try to explain the connection between SIgAD and autoimmunity (77, 126). For example, as described above, the HLA 8.1 haplotype is commonly related to different autoimmune diseases (i.e. type 1 diabetes and rheumatoid arthritis) and SIgAD (112–116, 131, 132). Also, both SIgAD and autoimmune disorders show familial clustering (58, 131, 133) and therefore, the increased frequency in autoimmune disorders might suggest a possible common genetic component in SIgAD and autoimmunity.

## Pathogenesis of SIgAD

### IgA Class-Switch Recombination

The large variability in clinical manifestations in SIgAD individuals suggests that there might be multiple and different causal mechanisms involved in the development of SIgAD. On the molecular and cellular level SIgAD has been associated with a defective terminal differentiation of membrane-bound IgA+ plasma blasts into IgA secreting cells, and this results in defect in the survival of IgA-secreting plasma cells, different T cell abnormalities, an impaired cytokine network, and a defect in the IgA class switching process (121, 134–136). IgA+ memory B cells (CD27+) in the intestine originate mainly from TI responses and are characterized by high IgA2 usage and increased reactivity to intestinal bacteria. In SIgAD patients a reduction in IgA+ memory B cells was found associated with no defect in class switching and affinity maturation. Therefore, it is suggested that SIgAD patients have defective regulation of the IgA response due to reduced Th1 and Th17 cells and increased blood concentrations of TGF-β1, BAFF and APRIL (137). In addition, increased numbers of CD27+IgA+ memory B cells were found in a cohort of children (n=729) having ever showed a food allergic reaction over the first 10 years of their life. Food‐allergic sensitization was associated with 22.5% higher IgA+ but not IgG+ or IgE+ memory B cells, which is explained by the fact that IgE+ B cells are more involved in current and mostly severe atopic disease (138). However, it is widely accepted that the main defect in individuals with SIgAD is a defect in the maturation from an IgA expressing (IgA+) B cell into an IgA secreting plasma cell that might be related to the process of alternative splicing of mRNA in IgA-secreting B cells and this results in the generation of membrane bound IgA+ B-cells but not secreted IgA antibodies (2–4, 6, 27, 82, 93). Although sometimes doubted, more work has substantiated this prevailing explanation of the underlying molecular mechanism of IgA deficiency (93, 94). In addition, some gene mutations, like a loss-of- function mutation in TACI, appear to be associated with SIgAD (106). TACI has a crucial role in the generation of humoral immunity, as TACI is involved in the activation of transcription factors like NFAT, AP-1 and NF-κ-B, T cell independent antibody responses, isotype switching and the maintenance of antibody-secreting cells (3, 89, 117, 139).

The B cell activating factor (BAFF) is produced in a membrane bound and secreted form, while the proliferation-inducing ligand (APRIL) is only produced in a secreted form. BAFF can bind to three different receptors: BAFF-R (BAFF-receptor), TACI, and to BCMA (B cell maturation-antigen). APRIL can bind to TACI and BCMA but not to BAFF-R. BAFF binding to BAFF-R expressed on B-cells, activated T-cells and Tregs induces survival and upregulation of co-stimulatory molecules enhancing thereby T-cell dependent isotype switching to IgA. BAFF and APRIL binding both to TACI expressed on B-cells, plasma blasts and macrophages induce isotype switching to IgA in a T-cell independent manner. BAFF and APRIL binding to BCMA which is expressed on plasma cells enhances plasma cell survival (3, 85–87, 117). Alternatively, a functional deficiency of cytokines like IL-4, IL-6, IL-7 and IL-10 might be causally related to the inability of B cells to differentiate into IgA-secreting plasma cells even when they are switched to IgA on the molecular level.

### IgA Production Pathway and Treg Cells

Recently, Bronson et al. (140) performed a meta-analysis on GWAS-based studies in which the affected pathways leading to IgA deficiency are described. This analysis showed an association of several genes within the Kyoto Encyclopedia of Genes and Genomes (KEGG) pathway “Intestinal Immune Network for IgA production” and with “T regulatory (Treg)” genes. This KEGG pathway analysis contained the following genes with a P value <0.05 in the IgA deficiency meta-analysis: genes encoding inflammatory and immunoregulatory cytokines (IL-2, IL-4, IL-6, IL- 10, IL-15, APRIL, BAFF), genes encoding B and T cells surface molecules (TACI, CD40, IL-15R, CD28, ICOS, ICOSLG), genes encoding gut-homing receptors on lymphocytes (ITGA4, CCR9, MADCAM-1, CXCL12, CCL28, LTβR, MAP3KI4) and genes encoding a DNA deaminase (AID) which is required for B cell class switch recombination. These data suggest that several of these genes involved in IgA production might contribute to the development of SIgAD (117, 139, 140).

Not only an association with the IgA production pathway was found, but also with the formation and functioning of Tregs. Tregs are present in substantial numbers in the intestinal mucosa and produce cytokines, including TGFβ and IL-10, which are important in IgA production (117, 140). Cong et al. (141) found that depletion of Treg cells decreased the formation of IgA+ B cells, total IgA and hence the intestinal IgA responses. In addition, it was shown that repletion of T cell-deficient mice with Tregs increased total intestinal IgA production. Based on these findings it was suggested that Tregs are major helpers for induction and maintenance of B-cells eliciting a T cell dependent IgA response in the intestinal mucosa. Any deficiency or polymorphisms in IL-10 or reduction in the number or activity of Tregs might thus contribute to the development of IgA deficiency. However, a defect in Tregs would probably cause many more immunological problems, because of their wide and important role. Local intestinal mucosa problems with Tregs, could then result in SIgAD only. However, further research is necessary into the possible association between Tregs and SIgAD.

## Epigenetics and Environmental Factors

### Epigenetic Modification of TNFRSF13 in SIgAD

In addition to the putatively involved genes and genetic abnormalities in SIgAD, environmental factors, including infections, can modify the transcriptional activity of these genes contributing to further disease development. In particular, the TNFRSF13 gene was shown to be modulated by epigenetic influences and thereby contributing to SIgAD. The main mechanism on which the epigenetic changes are based is the altered methylation state of the TNFRSF13B and TNFRSF13C genes which correlates to the expression levels of mRNA for TACI and BAFF- R and the presence of IgA levels, respectively. In dogs suffering from inflammatory bowel disease (IBD) associated with significantly decreased levels of IgA, the expression levels of IgA were associated with the DNA methylation state of the TNFRSF13B and TNFRSF13C genes and the mRNA expression levels of TACI and BAFF-R (141). The observed decrease in the mRNA expression of TACI and BAFF-R in the duodenal mucosa, were complemented with a hypermethylation of CpG islands in the genes of TNFRSF13B and TNFRSF13C, respectively. The degree of methylation of TNFRSF13B and TNFRSF13C showed a significant negative correlation with the mRNA expression level of TACI and BAFF-R in these canine B cells in the duodenum. Similarly, a negative correlation was observed between the methylation status of TNFRSF13B and serum IgA levels, while at the same time a positive correlation was found between the mRNA expression levels of TACI and serum IgA levels. However, these authors failed to find any significant correlation with BAFF-R and only further research can explain these complex findings (141).These results thus show that mRNA expression of TACI (and probably also BAFF-R) is regulated by the methylation degree of their corresponding genes in B cells of dogs. Whether this is also the case in humans with SIgAD, needs to be further investigated. This might be relevant as dogs with low IgA levels do show a very similar disease phenotype as humans with IgA deficiency. Furthermore, several genetic abnormalities have already been found in the TNFRSF13B gene in some individuals with CVID and IgA deficiency (91, 93), pointing to a role of TNFRSF13B in the development of IgA deficiency (and CVID) in some individuals. When correlations between TNFRSF13B (and TNFRSF13C), TACI (and BAFF-R), and IgA levels would be found in individuals with SIgAD, this would provide evidence for epigenetics being crucially involved in the development of SIgAD. Potentially, modulation of the expression of TACI and BAFF-R *via* increase in demethylation could aid in diagnostics and provide a rationale for novel immunotherapeutic treatment.

### Gut Microbiota Influence on IgA Levels

The gastrointestinal tract, being the largest mucosal surface area of the body, contains the most diverse microbiota composition (142). By using germ-free (GF) animals it was shown that the gut microbiota influences IgA levels, and the number and cellularity of gut mucosa associated Peyers Patches (PP) (143–145). GF mice are characterized by a decrease in the number of plasma cells and a decrease in circulating IgA levels. In addition, Bauer et al. found that the spleen of GF mice contains fewer and smaller germinal centers compared to normal mice and thereby a decrease in fully mature B cells and number of sIgA producing B cells (145). However, further research is necessary to prove this hypothesis.

In patients with SIgAD a reduced diversity in microbiota composition or dysbiosis was observed in the intestines, although this did not lead to profound changes in the composition of the fecal microbiota (146). Compared to healthy controls, the lack of IgA antibodies was correlated to the partly compensation by IgM antibodies, the shift from commensal bacteria to more pro-inflammatory microbial species, and the presence of activated Th17 cells related to the induction of inflammation in the intestinal mucosa (147). At the systemic level, IgG antibodies are found in SIgAD patients that are specific for bacterial and display anti-inflammatory activity (148). These results suggest that secretory IgA antibodies control the composition of the gut microbiota and ensure a stable and non-inflammatory condition in the gastrointestinal mucosa. Specific microbiota, including segmented filamentous bacteria (SFB) induce the development and rapid growth of Peyer’s patches, and the formation of other lymphoid tissues in the host (149, 150). By activating the intestinal immune system, a drastic increase in fecal concentration of sIgA was observed while at the same time the number and activity of IgA secreting B-cells is increased (151, 152). Both T-cell dependent and T-cell independent production of sIgA occur also in SFB mono-associated mice (149, 150). The relevance of interaction between gut microbiota and the developing immune system is further illustrated by microbiota that induce mucosal Th17 cells in the gut and levels of IgA (153). *Bacteroides fragilis* is one of the more common bacterial strains in the human gut and supplementation influenced the intestinal microbial community by producing a biofilm associating with the gut epithelium (153). Despite its associations with disease, *B. fragilis* also promotes immune tolerance in the gut by expressing polysaccharide A, which drives regulatory T cells to produce IL-10, crucial for maintaining immune tolerance (153–155). Prolonged inflammation in a sick individual may give rise to B. fragilis strains that are distinct from those found in a healthy individual. A positive association can be seen in allergy for peanuts and tree nuts with Bacteroides fragilis, increased the abundance of butyrate-producing bacteria, which in turn suppress the inflammatory responses triggered by Th2 cytokines (148–150). There is an individual heterogeneity in strains of B fragilis which might be due to reflect adaptation to diet- or host-derived glycans (153).Lastly, the short chain fatty acid butyrate, which is produced by multiple different bacteria that ferment dietary fibers, can indirectly induce Tregs (153–155). These examples thus illustrate the importance of the microbiota in relation to the development and the function of the gut-associated mucosal immune system and the local production of IgA (156). Since the gut microbiota is so important, IgA deficiency might also result from a lack of relevant microbiota required for triggering IgA production. If indeed the microbiota play a role in IgA productions, the question which specific microorganisms play this role and can potentially be used as treatment needs to be further investigated.

### Gender, Age and Seasonal Effects on IgA Deficiency

Not only gut microbiota seems to influence IgA levels, but also other factors might influence IgA levels. For example, Weber-Mzell et al. (157) have tested a group of 7293 healthy Caucasians (5020 men and 2264 women) and showed the influence of seasons, age and/or gender on serum IgA levels. In addition, they found that men have a higher prevalence of serum IgA deficiency and that the prevalence of serum IgA deficiency is lowest in winter. However, an explanation for these observations is lacking. In addition, there are studies that have not found any seasonal influence (158, 159). Because of the varying results regarding seasonal influence, it is likely that the influence of the seasons is small and contributes little to the development of IgA deficiency. Probably not only the seasonal influence is small, but also the influence of gender and age.

## Putative SIgAD Treatments

### Current Treatment

Currently, no specific treatment for patients with clinical symptoms of SIgAD is available. The treatments used are limited to the management of associated diseases, because replacement of IgA is not possible (3). Thereby, the question remains if replacement of IgA will be beneficial, because IgA has a relatively short half-life of 6-7 days in circulation. The type of treatment which is used depends on the (severity of the) clinical symptoms of an individual. Treatments that are used are for example, prophylactic antibiotic therapy to decrease the risk of infections (160, 161) and intravenous or subcutaneous administration of gammaglobulins which aims to increase (protective) IgG antibody levels. Patients with SIgAD displaying recurrent upper respiratory tract infections may benefit from at least a 6-month treatment with prophylactic antibiotics. Children with SIgAD that display seasonal patterns of infections in the winter period may benefit from receiving prophylactic antibiotics. Despite the use of prophylactic antibiotics, some patients with SIgAD continue to have recurrent infections which can be treated with intravenous immunoglobulin replacement therapy although this treatment remains controversial. IgG subclass levels should be monitored in children around one year of age and during further development and including specific IgG subclass responses to selected polysaccharide and protein antigens. This allows to determine whether these children could benefit from treatment with intravenous immunoglobulin preparations, although this has not been described yet.

### Retinoic Acid and Vitamin D

It is likely that SIgAD is based on a defect in the process of IgA class switching and therefore a hypothetical treatment based on retinoic acid (RA) is proposed. RA is a major vitamin A metabolite and high levels of RA can promote B cell class switching to IgA, and thereby increase the IgA production potential (162). However, whether RA directly influences class switching (163) or enhances the proliferation and differentiation of switched IgA plasma blasts (164, 165) remains to be established. It also appears that RA synergizes with other factors in the gut (147) that might promote IgA production. Next to this, RA might even be used in combination with vitamin D, because vitamin D induces Tregs and Tregs might also be involved in the development of SIgAD. In summary, RA is somehow positively involved in IgA production and might perhaps (in combination with vitamin D) be used as treatment for SIgAD. However, no literature is available yet on this subject.

### Gut Microbiota: Bacteroides Fragilis and Butyrate

The gut microbiota might be used as a treatment for immunodeficiencies or other immune-related diseases because of their crucial role in the development and functioning of the mucosal immune system. The microbiota and the intestinal immune system display a high level of tolerance which is mediated by high numbers of Treg that are also implicated in the induction and maintenance of intestinal IgA B cell responses. Similarly, SIgAD is associated with Tregs genes based on their pathway analysis (117, 140). In addition, it was described that *Bacteroides fragilis* and the short chain fatty acid butyrate, which is produced by a variety of bacteria, induce Tregs formation (156-158. Together these observations suggest that *Bacteriodes fragilis* (or a closely related species that does occur in humans) and the short chain fatty acid butyrate could be potential candidates for the treatment of SIgAD *via* Treg induction. Induction of increased numbers and functioning of Tregs might potentially provide a treatment option for SIgAD (166–168)

### Novel Treatment Option: TNFRSF13

Defective IgA class switching might still be the main molecular defect in SIgAD. TACI is involved in T cell independent class switching and hence, a mutation in TACI could be involved in SIgAD. Several studies have found mutations in TNFRSF13B and abnormalities in the methylation of TNFRSF13B which support the involvement of TACI. Polymorphisms in TNFRSF13B are likely to affect parts of the innate and the adaptive immune system. It was previously suggested that whenever a TNFRSF13B mutation and an immunodeficiency profile were present, these patients also suffered from SIgAD. In addition one SIgAD patients was indeed identified that also had a TNFRSF13B mutation (169, 170). Recently, however, the presence of at least one mutation in the TNFRSF13B gene was found in 13% of SIgAD patients (171). Most mucosal IgA is generated from T-cell independent antibody generation which is driven by TNFRSF13 that promotes the differentiation of plasma cells (172). TNFRSF13B polymorphisms are considered to be associated with SIgAD (3) and these polymorphisms are maintained in population by the adaptive capacity of microbiota to mucosal IgA antibodies (173). Therefore, TNFRSF13 appears to be a good candidate gene in the development of SIgAD and inducing the expression of TACI *via* demethylation of the relevant DNA sites could provide a rational target for novel therapeutics (174).

As also Tregs might be involved in the development of SIgAD, the combination of these two possible candidates could provide a novel treatment can be obtained by being based on the combination of the vitamin A derivate RA, which can improve IgA production and vitamin D, which can induce Tregs. The major advantage of this treatment is the fact that this treatment is specific for SIgAD, because RA selectively acts on IgA production. Other proposed treatments, for example based on the use of IL21 or combinations of activation of CD40 by CD40L or agonistic anti-CD40 antibodies, IL-4 and IL10 (54) are less isotype-specific as these cytokines act on more immune mechanisms besides IgA production. Such a treatment is safe and easy to administer because vitamin A is already present in a great variety of food, like different vegetables, and vitamin D can be obtained from the sun exposure or by supplements. However, at this point in research, it is known that RA positively influences IgA production of normal functional B cells. The question remains if RA would also increase IgA production in defective B cells of individuals with SIgAD and thus research needs to be done to determine this.

## Conclusion

In conclusion, selective IgA deficiency is increasingly recognized as a condition which involves specific aberrations in both the innate and the adaptive immune response. TACI might be a novel therapeutic candidate involved in the development of SIgAD *via* a defect in IgA class switching. TACI is involved at the very beginning of the IgA production pathway, leading to the secretion of IgA. Because of this early involvement of TACI many other factors could influence the IgA production pathway, including Tregs, HLA genes and the methylation status of selected genes. The contribution of each of these factors, many of which belong to the innate immune system, can contribute to the variation in the clinical manifestations and this involvement needs to be investigated further. In addition, so far only associations rather than causal relations between susceptibility genes and SIgAD are described and further knowledge on the underlying genetic causes and the molecular defect in IgA class switching is needed. This knowledge might set the stage for the rational design of novel therapeutics and treatments.

## Author Contributions

HS and JL conceived the subject and structure of the review. DO and JZ performed most of the literature search. All authors agree to be accountable for the content of the work. All authors contributed to the article and approved the submitted version.

## Funding

JZ was funded by a scholarship from the Chinese Scholarship Council, nr 201803250028 and currently on a sandwich PhD project from the Chinese Academy of Agricultural Sciences and Wageningen University.

## Conflict of Interest

The authors declare that the research was conducted in the absence of any commercial or financial relationships that could be construed as a potential conflict of interest.
